# Prevalence of asthma in preterm and associated risk factors based on prescription data from the Korean National Health Insurance database

**DOI:** 10.1038/s41598-023-31558-z

**Published:** 2023-03-18

**Authors:** Kwanghoon Kim, Ji Young Lee, Yoo-Mi Kim, Geena Kim, Eun-Hee Kim, Byoung Kook Lee, Hyejin So, Yoowon Kwon, Jeongmin Shin, Minji Kim

**Affiliations:** 1 Department of Pediatrics, Busan Paik Hospital, Inje University College of Medicine, Busan, Korea; 2 Department of Pediatrics, Chuncheon Sacred Heart Hospital, Hallym University School of Medicine, Chuncheon, Korea; 3 Department of Pediatrics, College of Medicine, Chungnam National University, Chungnam National University Sejong Hospital, Sejong, Korea; 4 Department of Pediatrics, College of Medicine, Eunpyeong St. Mary’s Hospital, The Catholic University of Korea, Seoul, Korea

**Keywords:** Preterm birth, Epidemiology

## Abstract

We retrospectively analyzed National Health Insurance claims data (January 2002–December 2018) to determine the asthma prevalence and risk factors among preterm infants born in Korea. Patients with asthma were defined as those with a history of asthma medication prescriptions at least twice per year with International Classification of Diseases, Tenth Edition codes J45 and J46. We enrolled 99,139 preterm infants. The prevalence of asthma among preterm and term infants was 32.7% and 26.9%, 21.2% and 19.1%, 6.7% and 5.9%, 2.0%, and 1.6%, and 2.4% and 1.6% at 2, 5, 10, 15, and 16 years of age, respectively. The relative risk (RR) of asthma in preterm infants was 1.1-fold that in female preterm infants. The RR of asthma medication prescriptions for infants with extreme prematurity was 1.92-fold that of infants with moderate/late pre-term status. Among preterm with bronchopulmonary dysplasia (BPD) and respiratory distress syndrome (RDS) without comorbidities, the RRs for the number of asthma medication prescriptions were 1.34 and 1.06, respectively. This study revealed a higher prevalence of asthma among preterm infants than that in term infants. Male sex, extreme prematurity, BPD, and RDS were identified as risk factors for asthma medication prescriptions in preterm infants.

## Introduction

The number of births in developed countries has decreased due to low fertility; however, birth rates of premature infants (< 37 completed weeks of gestation) are increasing. The reasons for the increase in preterm births include older age at pregnancy and increased performance of infertility procedures^1^. In most developed countries, the incidence of preterm birth ranges from 5 to 12%^2^. Neonatal intensive care management has advanced over the last 30 years thereby increasing survival rates among premature newborns^3^. However, chronic respiratory diseases are common sequelae of preterm birth in later life, especially among extremely premature neonates or those with bronchopulmonary dysplasia (BPD)^4^.

Preterm infants are more susceptible to respiratory infectious diseases and require hospitalization more often than term infants. A preterm cohort study conducted in France revealed that 47.3% of infants were readmitted at least once within 9 months of birth, and 55% of them were admitted for respiratory disorders^5^. A US cohort study reported that asthma and respiratory symptoms identified by parents within the previous 12 months were twice as common in preterm infants compared with the control group^6^. Respiratory symptoms in ex-preterm infants generally become mild by the time they reach school age^7^. However, some preterm infants have persistent obstructive lung disease until adulthood^8^. Respiratory symptoms present as chronic obstructive airway disease, characterized by recurrent episodes of wheezing, and decreased forced expiratory volume in 1 s in later in life^7^. According to the European Community Respiratory Health Survey, adults with a medical history of BPD were twice as likely to complain of wheezing and three times as likely to use asthma medication compared with adults in the term control group^9^.

Korea was recently included among countries with the lowest birth rates (0.977 in 2018) worldwide^1^. However, Korea has high survival rates for premature infants. According to the Korean Neonatal Network findings, the survival rate of very low birth weight infants is 86%^10^. Therefore, the prognosis of preterm infants after survival is essential. Nevertheless, no reports have been published on the prognosis of premature infants with respiratory diseases in Korea. In Korea, all medical institutions are monitored by the National Health Insurance (NHI) program, which is run by the government. All medical statuses and healthcare usages are recorded by the NHI. Using the NHI database, we aimed to determine the prevalence of asthma based on asthma medication prescriptions, frequency of asthma treatment among premature infants, and asthma-related risk factors associated with prematurity.

## Results

### Asthma prevalence

**Figure 1:**
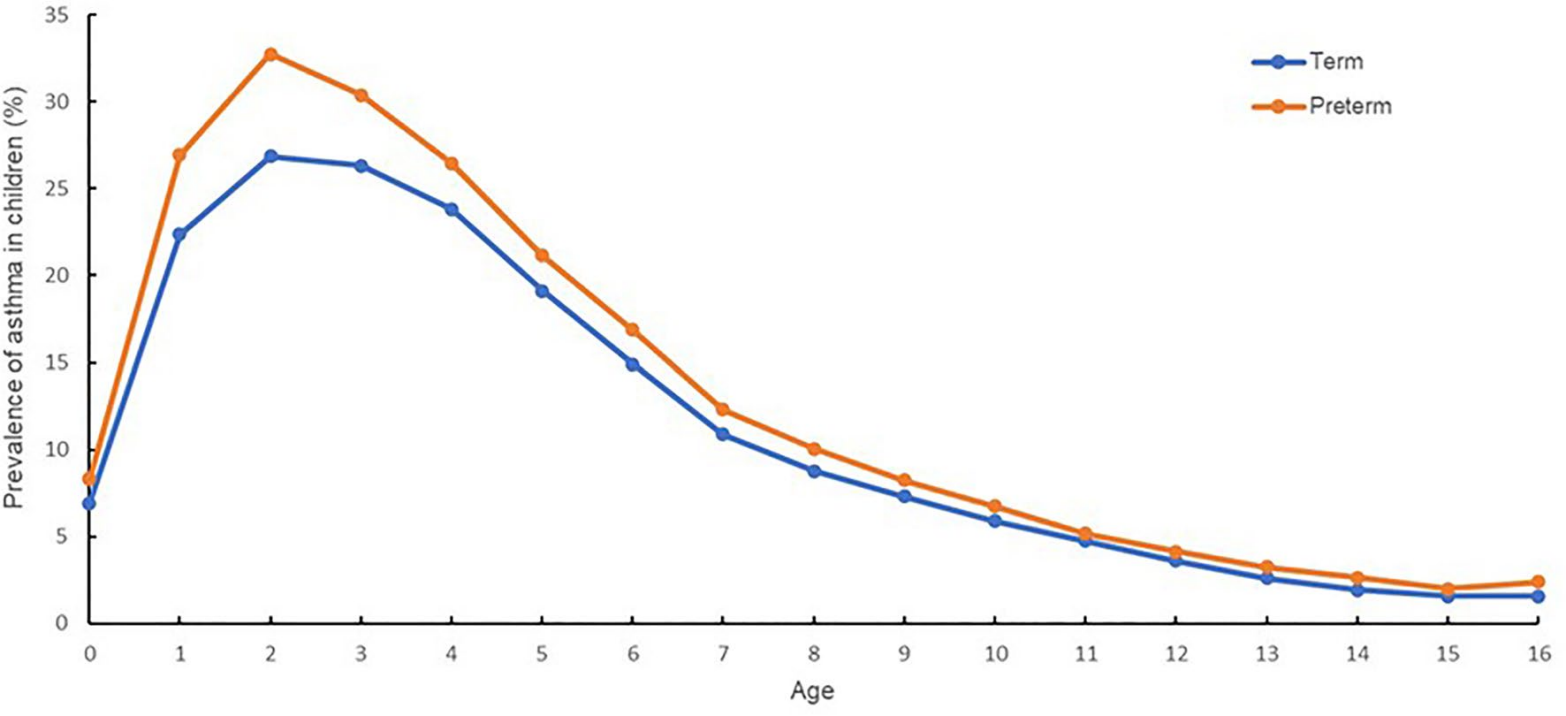
Asthma prevalence in preterm and term infants stratified by age. An orange line represents asthma prevalence in preterm infants stratified by age. A blue line represents asthma prevalence in term infants stratified by age.

The number of term infants born in Korea in 2002 was 500,546, gradually decreasing to 331,722 in 2018. From 2002 to 2018, the number of children registered as preterm infants within the first year of life was 99,139. Premature infants born between 2002 and 2018 were followed up annually to estimate the prevalence of asthma compared with the general birth population. Figure 1 shows the prevalence of asthma according to age among premature and term infants. The prevalence of asthma among the general population for various ages was 6.9% (for less than 12 months), 22.4% (1 year), 26.9% (2 years), 26.3% (3 years), 23.8% (4 years), 19.1% (5 years), 14.9% (6 years), 10.9% (7 years), 8.8% (8 years), 7.3% (9 years), 5.9% (10 years), 4.8% (11 years), 3.6% (12 years), 2.6% (13 years), 1.9% (14 years), 1.6% (15 years), and 1.6% (16 years). The prevalence of asthma among preterm infants of various ages was 8.3% (less than 12 months), 26.9% (1 year), 32.7% (2 years), 30.4% (3 years), 26.5% (4 years), 21.2% (5 years), 16.9% (6 years), 12.3% (7 years), 10.1% (8 years), 8.3% (9 years), 6.7% (10 years), 5.2% (11 years), 4.1% (12 years), 3.3% (13 years), 2.6% (14 years), 2.0% (15 years), and 2.4% (16 years). When the asthma prevalence for various ages were compared, the relative risk (RR) was higher for patients with asthma by approximately 1.1–1.5 times (Table 1).

**Table 1:** Asthma prevalence stratified by age.

Age (yr)	Term			Preterm			Relative Risk
	Population	Asthma	Prevalence (%)	Population	Asthma	Prevalence (%)	
0	7,264,218	503,212	6.93	99,139	8262	8.33	1.20 (1.18–1.23)***
1	7,264,218	1,623,888	22.35	99,139	26,706	26.94	1.21 (1.19–1.22)***
2	6,942,661	1,865,390	26.87	88,974	29,130	32.74	1.22 (1.20–1.23)***
3	6,583,351	1,731,872	26.31	78,573	23,889	30.40	1.16 (1.14–1.17)***
4	6,184,622	1,474,373	23.84	68,204	18,046	26.46	1.11 (1.09–1.13)***
5	5,772,663	1,103,919	19.12	57,981	12,261	21.15	1.11 (1.09–1.13)***
6	5,361,207	798,900	14.90	48,802	8241	16.89	1.13 (1.11–1.16)***
7	4,926,781	536,313	10.89	40,327	4963	12.31	1.13 (1.10–1.16)***
8	4,477,049	393,457	8.79	32,100	3234	10.07	1.15 (1.11–1.19)***
9	4,033,500	295,744	7.33	25,631	2115	8.25	1.13 (1.08–1.17)***
10	3,603,316	212,519	5.90	19,313	1300	6.73	1.14 (1.08–1.21)***
11	3,173,885	150,854	4.75	14,269	740	5.19	1.09 (1.01–1.17)*
12	2,720,664	98,489	3.62	10,150	420	4.14	1.14 (1.04–1.26)**
13	2,276,632	59,301	2.60	6685	217	3.25	1.25 (1.09–1.42)**
14	1,859,564	35,787	1.92	4455	117	2.63	1.36 (1.13–1.64)**
15	1,428,298	22,368	1.57	3183	64	2.01	1.29 (0.99–1.64)
16	969,159	15,240	1.57	2077	50	2.41	1.53 (1.14–2.02)**
17	500,546	7,074	1.41	930	9	0.97	0.68 (0.31–1.30)

*p < .05; **p < .01; ***p < .001.

### Number of Asthma medication prescriptions

**Figure 2:**
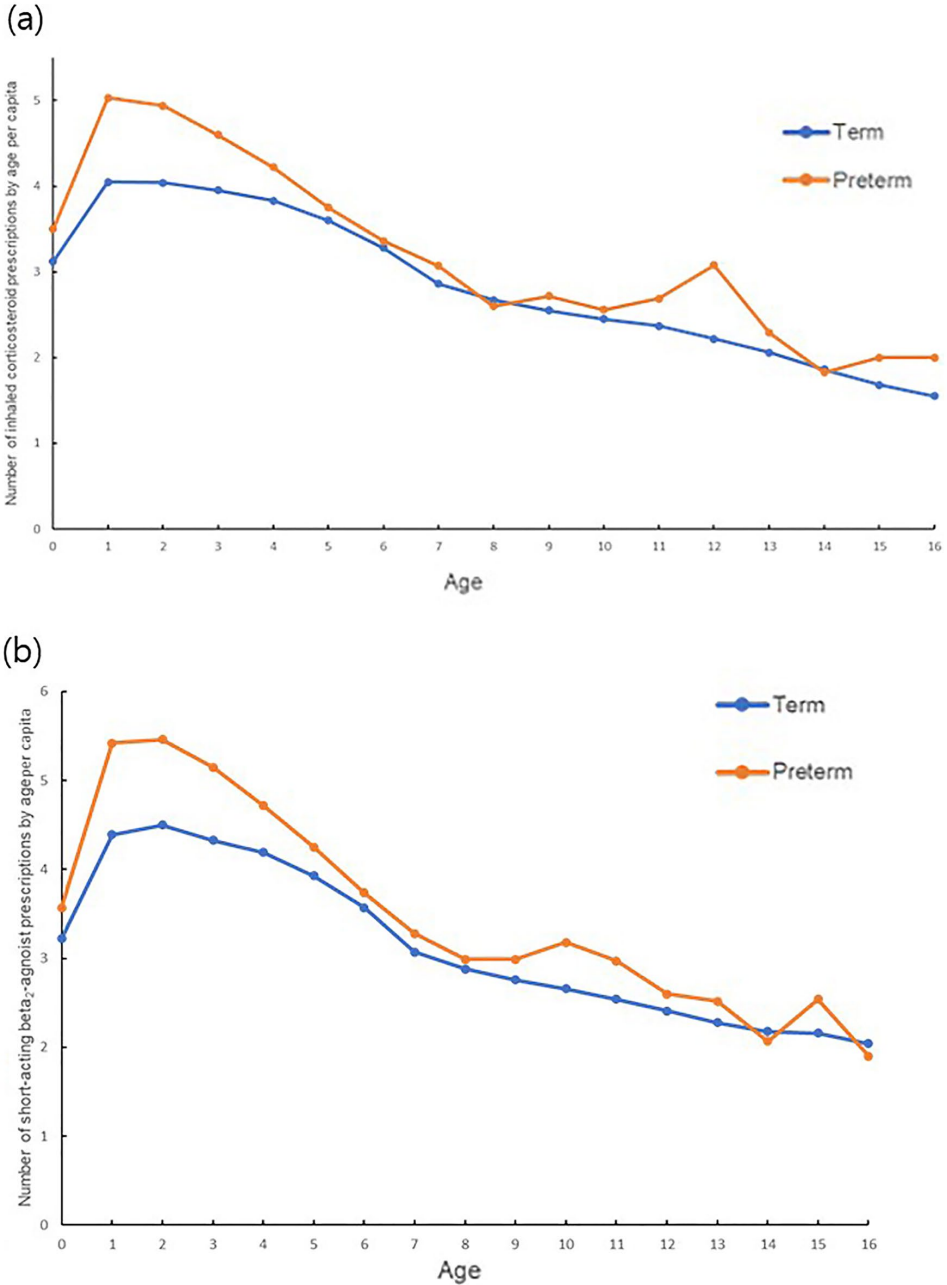
(**a)** Number of inhaled corticosteroid prescriptions per asthma patient for term and preterm infants by age per capita. (**b)** Number of prescriptions for short-acting β2 agonists per asthma patient for term and preterm infants stratified by age per capita. The number of inhaled medications prescribed to asthmatic patients are expressed by the orange and blue lines for preterm and term infants, respectively.

The number of prescribed medications for patients with asthma indirectly reflects the asthma severity. We compared the number of prescriptions for inhaled corticosteroids (ICSs) and short-acting β_2_ agonists (SABAs) between term and preterm asthmatics. ICSs and SABAs are necessary medications in asthma treatment that can be used from a young age through adolescence. Figure 2 shows the number of asthma medications per patient according to age. Figure 2a shows the frequency of ICS prescriptions per asthma patient among term and premature infants; Fig. 2b shows the number of SABA prescriptions per asthma patient among the term and premature infants by age. These figures show that ICSs and SABAs were prescribed more frequently to premature infants than to term infants in most age groups.

**Figure 3:**
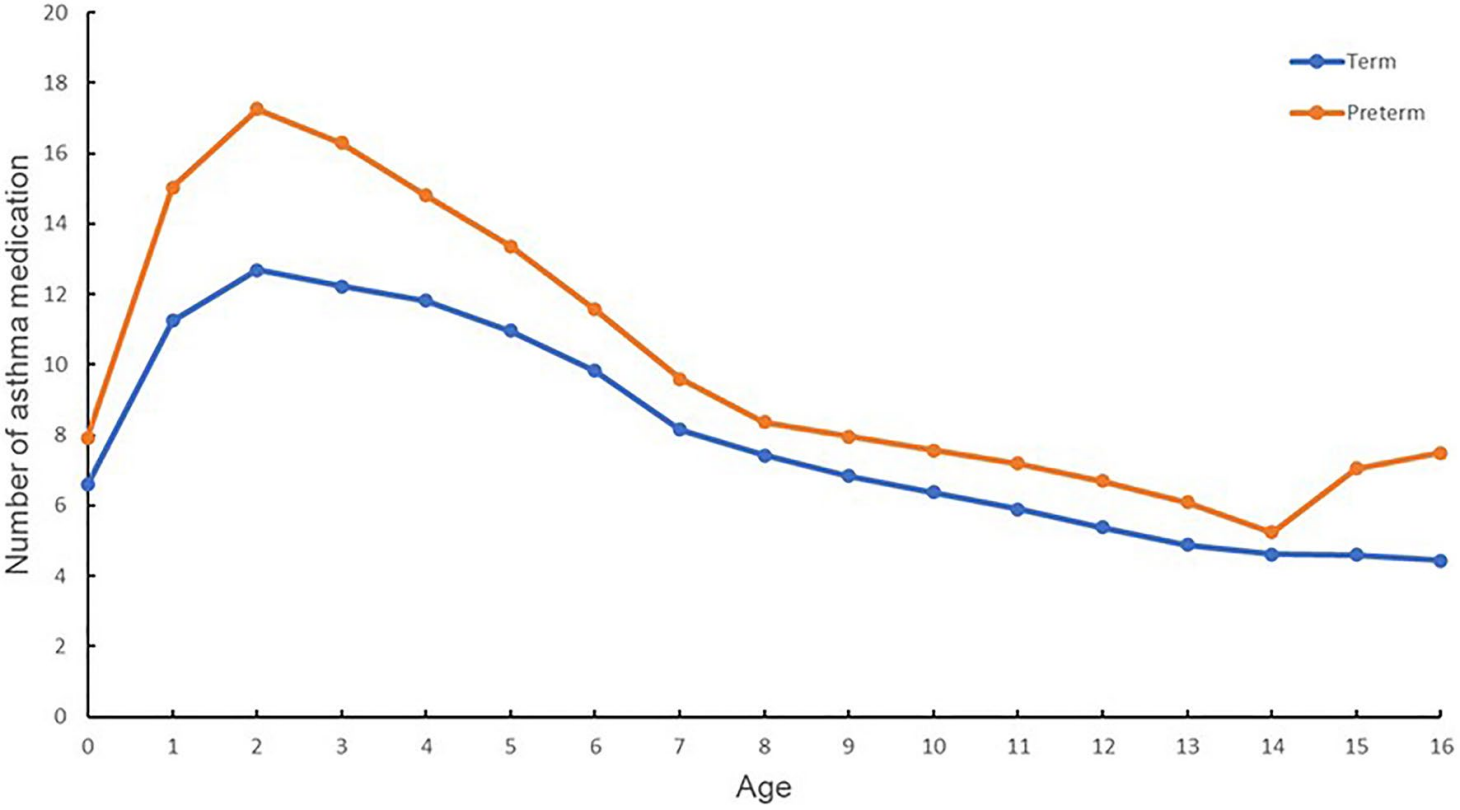
Number of asthma-related medications prescribed per patient stratified by age. An orange line depicts the number of asthma-related medications prescribed to preterm infants, stratified by age. A blue line represents the number of asthma-related medications prescribed to term infants, stratified by age.

We compared the number of asthma-related medication prescriptions among patients with asthma in term and preterm infants. The number of asthma medication prescriptions included not only those for acute asthma attacks but also medications for asthma control and management. Figure 3 shows the number of asthma-related medication prescriptions per patient stratified by age group. The number of hospital visits for asthma and prescribed asthma-related medications at 1 year of age was 11.25 per patient for term infants compared with 15.03 for premature infants. At 2 years of age, the number of hospital visits for asthma and prescriptions for related medications were 12.68 per patient for term infants and 17.25 for preterm infants. At the age of 5 years, the number of prescriptions for asthma-related medications was 10.97 for term infants and 13.38 for premature infants. At the age of 10 years, the number of prescriptions for asthma-related medications was 6.37 for term infants and 7.57 for preterm infants. At the age of 15 years, the number of prescribed asthma-related medications was 4.6 for term infants and 7.05 times for preterm infants.

### Asthma-related drug prescription rates and risk factors in preterm infants

An additional analysis was performed to determine the risk factors for asthma in preterm infants (Table 2). Among the preterm infants, the number of all asthma-related prescriptions according to demographic factors or comorbidities was evaluated. Preterm male infants with asthma, relative to their female counterparts, had a significantly increased risk (RR: 1.1, 95% confidence interval [CI] = 1.09–1.12) for the prescription of asthma medication. Preterm infants with extreme prematurity and other preterm infants had a 1.92-fold (95% CI = 1.82–2.03) and 1.57-fold (95% CI = 1.51–1.63) increase in prescriptions for asthma, respectively, relative to that of moderate/late pre-term infants. A 1.80-fold increase was observed in the risk for prescriptions for asthma medications in infants with extremely low birth weight (RR: 1.80, 95% CI = 1.68–1.94) and a 1.43-fold increased risk in infants with very low birth weight (RR: 1.43, 95% CI = 1.34–1.54) relative to that of infants with low birth weight. Preterm infants with BPD had a 1.34-fold increased risk for prescriptions for asthma medications (RR: 1.34, 95% CI = 1.29–1.39). Respiratory distress syndrome (RDS) led to a 1.06-fold increased risk (RR: 1.06, 95% CI = 1.04–1.08) compared with infants without those diseases. Preterm infants with intrauterine growth retardation, those who were large for gestational age (GA), and those who were small for GA (SGA) had a decreased risk (RR: 0.68, 95% CI = 0.59-0.77; RR: 0.67, 95% CI = 0.50–0.88, RR: 0.60, 95% CI = 0.54–0.67) of asthma relative to preterm infants without comorbidities.

**Table 2:** Asthma-related drug prescription rates per person diagnosed with asthma and risk factors in preterm infants.

	Preterm			Relative risks
	Population	With asthma	Incidence rate	
Sex				
Male	326,898	80,522	24.63	1.11 (1.09–1.12)***
Female	268,237	59,710	22.26	Reference
Preterm				
Extreme preterm	6292	1589	25.25	1.92 (1.82–2.03)***
Very preterm	16,177	3336	20.62	1.57 (1.51–1.63)***
Moderate/late preterm	71,366	9387	13.15	Reference
Weight				
Extremely low birth weight	4298	1135	26.41	1.80 (1.68–1.94)***
Very low birth weight	6001	1260	21.00	1.43 (1.34–1.54)***
Low birth weight	16,756	2452	14.63	Reference
Preterm without comorbidities	595,135	140,232	23.56	Reference
Small for gestational age	2264	322	14.22	0.60 (0.54–0.67)***
Large for gestational age	336	53	15.77	0.67 (0.50–0.88)**
Intrauterine growth retardation	1549	247	15.95	0.68 (0.59–0.77)***
Bronchopulmonary dysplasia	9009	2844	31.57	1.34 (1.29–1.39)***
Respiratory distress of newborn	43,841	10,983	25.05	1.06 (1.04–1.08)***

*p < .05; **p < .01; ***p < .001.

## Discussion

To our knowledge, this is the first study in Korea to highlight the importance of respiratory disease prognosis after preterm birth in later life. This study showed that preterm infants in Korea are at a significantly higher risk of developing asthma based on asthma diagnostic code and asthma-related medication prescriptions. In addition, preterm asthmatics were also prescribed asthma treatment more frequently than infants born term. We also found that asthma medications were prescribed more frequently to preterm asthmatic patients who were also extremely preterm and male, with extremely low birth weight, BPD, or RDS.

Most studies have reported the risk of chronic respiratory disorders among preterm infants. A meta-analysis reported that the incidence of childhood asthma among preterm infants was 1.37–1.71 times higher than that in term infants^11,12^. Structured questionnaires were collected from caregivers of preterm infants born before 29 weeks of gestation who visited a pediatrician at an outpatient clinic at 6 and 12 months of corrected age^13^. Overall, 27% of the patients had a cough, 20% had wheezing, and 3% had frequent wheezing. During the follow-up period, 14% of patients were prescribed bronchodilators, and 8% were prescribed steroids. Preterm survivors had more wheezing events, resulting in significantly impaired quality of life during adulthood^9^.

Several hypotheses have been proposed for the cause of wheezing in preterm infants. Pulmonary structural development was disrupted in preterm infants born without a sufficient period for lung maturation in utero^14^. Several studies have suggested that antenatal steroids may play a role in the development of asthma via the impairment of the hypothalamic-pituitary axis, immune dysfunction, and secondary hypertension^15^. Besides anatomical origins, several studies have proposed additional dynamic development in the airways of preterm born infants with an ongoing disease that may be caused by preterm birth or postnatal environmental factors^16,17^. One cohort study suggested that preterm birth is an independent risk factor for persistent biological stress via ongoing airway inflammation with rapid progression of telomere shortening^18^. Preterm birth also causes frequent injuries, including sepsis, respiratory infections, and hypoxia after birth^19^. Pathogenesis may contribute to the dose–response relationship between GA and chronic respiratory outcomes, including wheezing disorders.

Asthma is a heterogeneous disease characterized by inflammatory pathogenesis, bronchial hyperresponsiveness, and chronic airway obstruction. Whether the mechanism of asthma in preterm infants is the same as that in term infants is debatable. Several studies have shown that children born preterm have a lower incidence of atopic disorders over the long term^20,21^. Asthma patients born term is mainly induced by contact with allergens, however, asthma in preterm infants may be triggered by respiratory infections, reduced airway size, and decreased lung function^20^. Compared with term infants, preterm infants reportedly to secrete more respiratory cytokines, which may promote airway inflammation thereby predisposing them to asthma^22^. One study reported no evidence of steroid-sensitive inflammation within the lung assessed by fractional exhaled nitric oxide but suggested the possibility of increased systemic elastic turnover^9^. Several studies have reported that lung function in preterm infants decrease; changes include obstructive patterns, air trapping, and airway hyper-responsiveness, even in adults^9,14,23^. Consequently, preterm children should be evaluated for the development of asthma and receive aggressive treatment when detected.

In preterm infants, the risk factors for asthma include atopic dermatitis in infancy, allergic family history, antibiotic treatment within the first 3 years of life, and prematurity^24^. Similarly, male sex, prematurity, and BPD were associated with an increased risk of asthma among children born preterm in a previous study^25^. A previous study reported that female sex and SGA were associated with decreased risk of developing asthma^26,27^. The authors suggested that certain forms of fetal stress may result in accelerated development of the lungs, and maturation of the lungs is slower in men than in women. However, these results remain controversial. Some studies have also reported that intrauterine growth retardation increases bronchial hyperresponsiveness regardless of atopy tendency^28^.

This study has some limitations. First, the NHISS did not provide detailed clinical data and depended on physician registrations. The proportion of preterm births is estimated to be approximately 4.4–7.2% in Korea^29^; however, the proportion of registered preterm infants was 0.2–3.06%. Data on preterm births were limited due to the low registration rate of the diagnostic code and the low relative proportion of preterm infants. The diagnostic code registration rate was low in the early stages of establishing the national information data. Nevertheless, the registration rate increased as the national preterm infant care support system expanded. Preterm infants have an underlying medical history; therefore, the possibility of increased hospital resource utilization is also increased. In addition, a limitation exists in that the data were analyzed only when asthma treatment was performed in a medical institution in Korea. However, health claims data from the NHI are representative, as they cover approximately 98% of the Korean population^30^, and no additional operational definition was implemented to prevent selection bias. These are long-term data accumulated over 17 years of tracking, beginning with the conditions at birth, which can facilitate a better understanding of the overall health care of asthma patients. This study defined the diagnosis of asthma based on diagnosis codes and prescriptions for asthma-related drugs; however, this represents a limitation because this definition does not include detailed clinical information of the patient and radiological examination or pulmonary function test results. In actual clinical practice, there is a possibility that the diagnosis is incorrectly recorded, or asthma related drugs may be prescribed regardless of asthma diagnosis. To overcome errors in the diagnosis registration, characterizing asthma based on prescriptions for more than two asthma medications with a diagnosis of asthma is helpful. In addition, it is possible that preterm infants were diagnosed with asthma with a relatively more sensitive predictive value compared to term infants, because the former have a higher hospital utilization rate.

This study revealed a higher prevalence of asthma among preterm infants than that in term infants based on diagnosis code and asthma-related medication using the NHI database in Korea. Our findings also showed that preterm infants with asthma were prescribed ICSs and SABAs more frequently. Furthermore, preterm asthma patients were reportedly prescribed ICSs and SABAs, which are inhaled asthma-related drugs, more frequently than term asthma patients. Male sex, extreme prematurity, extremely low birth weight, BPD, and RDS were risk factors for asthma among preterm infants. The results of this study will guide pulmonary prognostication and follow-up strategies for preterm infants as well as directions for future studies on asthma prevention.

## Materials and methods

### Data sources and ethical approval

More than 98% of South Koreans are enrolled in the NHI program, which has a significant advantage in that the NHI database includes data for nearly the entire country's population^30,31^. These data have been used in big data clinical research to reflect real-world data^31,32^. The NHI program includes general demographic data, disease details, prescription information, and procedure or surgery details. The general demographic details include the patient's age, sex, dates of hospital visits, and disease diagnoses. The prescription information includes the drugs, number of prescription days, and quantity information. The disease diagnosis is registered based on the International Classification of Diseases (ICD)—10 codes, which is also the basis for extracting data for research. Korea's NHI established the NHI Sharing Service (NHISS) database to facilitate open access and distribution of national health information^33^. We studied newborns over a 17-year period from 2002 to 2018. The pediatric population with asthma between 2002 and 2018 was assessed using NHISS data by tracking birth-related factors (see Supplementary Fig. S1 online). This study adhered to the tenants of the Declaration of Helsinki and was approved by the institutional review board of Chungnam National University Sejong Hospital, Sejong, Republic of Korea (approval number: 2020-12-002-002). Institutional review board of Chungnam National University Sejong Hospital, Sejong, Republic of Korea waived the requirement for informed consent owing to the retrospective nature of the study and use of anonymized data.

### Study population and disease definitions

The pre-term infants were identified by the diagnostic codes for extreme prematurity (ICD-10: P07.2, GA < 28 weeks) and other prematurity categories (P07.3, 28 weeks ≤ GA < 37 weeks). Asthma was defined by the diagnostic code for asthma (ICD-10: J45–J46) in the primary and fourth order of secondary diagnoses, with asthma medication prescribed more than twice each year. Asthma medications included ICSs, long-acting β2 agonists, ICS/long-acting β2 agonists, SABAs, leukotriene receptor antagonists, anticholinergics, and xanthines (Supplementary Table S1 online). Through this retrospective population-based study, we determined the prevalence of asthma by age, asthma by age among pre-term infants, drug prescription frequency among asthma patients, and risk factors for asthma among premature infants. To identify the relevant risk factors for prematurity, data on GA classification (extremely preterm: GA < 28 weeks; very preterm; 28 weeks ≤ GA < 32 weeks; moderate/late preterm: 32 weeks ≤ GA < 37 weeks), body weight (extremely low birth weight, < 1000 g; very low birth weight, < 1500 g; and low birth weight, < 2500 g), BPD (ICD-10: P271), RDS in newborns (ICD-10: P220), and intrauterine growth retardation (ICD-10: P059) were analyzed. SGA (ICD-10: P051) and large for GA (ICD-10: P081) were defined as birthweights below the 10th and above the 90th percentiles, respectively.

### Statistical analysis

The number of newborns each year was determined using data from the Korean Statistical Information^34^. The annual asthma prevalence for each age was calculated by dividing the number of diagnosed asthma cases by the total number of persons with accumulated data in the NHISS and Statistics Korea databases. A two-sample t-test was performed to compare the significance of differences, as applicable. We compared the number of asthma medication prescriptions per person diagnosed with asthma among people born prematurely. The incidence rate ratio compared with that of the reference group was calculated to compare incidence rates among groups. Comparison groups were relatively determined, that is, in cases involving male patients, female patients were the reference. Infants with extreme preterm and very preterm were compared with infants with moderate/late preterm. Extremely low and very low birth weight infants were compared with low-birth-weight infants. SGA, large for GA, intrauterine growth retardation, and RDS in newborns were compared among premature infants without comorbidities. Statistical significance was set at a *P*-value of < 0.05. All statistical analyses were performed using SAS (version 9.4; SAS Institute Inc., Cary, NC, USA) and Stata (Version 17.0, College Station, TX, USA) software. All analyses were reported according to Strengthening the Reporting of Observational Studies in Epidemiology guidelines.

## Supplementary Information

Supplementary Information 1.

Supplementary Information 2.

## Data Availability

Although data are accessible from National Health Insurance (NHI) database, the access to data used in this study is provided only for the researchers who have applied for and have obtained permission. Further information is available on the online homepage of NHI Sharing Service (https://nhiss.nhis.or.kr). The data that support the findings of this study are available from the corresponding author, [M.K], upon reasonable request.

## References

[1] Lee JH, Youn Y, Chang YS (2020). Short- and long-term outcomes of very low birth weight infants in Korea: Korean Neonatal Network update in 2019. Clin. Exp. Pediatr..

[2] Chawanpaiboon S (2019). Global, regional, and national estimates of levels of pre-term birth in 2014: A systematic review and modelling analysis. Lancet Glob. Health.

[3] Gong A, Johnson YR, Livingston J, Matula K, Duncan AF (2015). Newborn intensive care survivors: A review and a plan for collaboration in Texas. Matern. Health Neonatol. Perinatol..

[4] Islam JY, Keller RL, Aschner JL, Hartert TV, Moore PE (2015). Understanding the short- and long-term respiratory outcomes of prematurity and bronchopulmonary dysplasia. Am. J. Respir. Crit Care Med..

[5] Lamarche-Vadel A (2004). Re-hospitalization in infants younger than 29 weeks' gestation in the EPIPAGE cohort. Acta Paediatr..

[6] Palta M (2001). Respiratory symptoms at age 8 years in a cohort of very low birth weight children. Am. J. Epidemiol..

[7] Damgaard ALB (2018). The increased purchase of asthma medication for individuals born pre-term seems to wane with age: A register-based longitudinal national cohort study. PLoS ONE.

[8] Vollsæter M, Røksund OD, Eide GE, Markestad T, Halvorsen T (2013). Lung function after pre-term birth: Development from mid-childhood to adulthood. Thorax.

[9] Gough A (2014). Impaired lung function and health status in adult survivors of bronchopulmonary dysplasia. Eur. Respir. J..

[10] Lee JH, Noh OK, Chang YS (2019). Neonatal outcomes of very low birth weight infants in Korean neonatal network from 2013 to 2016. J Korean Med Sci.

[11] Been JV (2014). Pre-term birth and childhood wheezing disorders: A systematic review and meta-analysis. PLoS Med..

[12] Jaakkola JJ (2006). Pre-term delivery and asthma: A systematic review and meta-analysis. J. Allergy Clin. Immunol..

[13] Greenough A (2005). Risk factors for respiratory morbidity in infancy after very premature birth. Arch. Dis. Child Fetal. Neonatal. Ed.

[14] Lum S (2011). Nature and severity of lung function abnormalities in extremely pre-term children at 11 years of age. Eur. Respir. J..

[15] Pole JD, Mustard CA, To T, Beyene J, Allen AC (2009). Antenatal steroid therapy for fetal lung maturation: Is there an association with childhood asthma?. J. Asthma.

[16] Sanchez-Solis M, Perez-Fernandez V, Bosch-Gimenez V, Quesada JJ, Garcia-Marcos L (2016). Lung function gain in pre-term infants with and without bronchopulmonary dysplasia. Pediatr. Pulmonol..

[17] Sanchez-Solis M, Parra-Carrillo MS, Mondejar-Lopez P, Garcia-Marcos PW, Garcia-Marcos L (2020). Preschool asthma symptoms in children born preterm: The relevance of lung function in infancy. J. Clin. Med..

[18] Hadchouel A (2015). Salivary telomere length and lung function in adolescents born very pre-term: A prospective multicenter study. PLoS ONE.

[19] Lahra MM, Beeby PJ, Jeffery HE (2009). Intrauterine inflammation, neonatal sepsis, and chronic lung disease: A 13-year hospital cohort study. Pediatrics.

[20] Goedicke-Fritz S (2017). Pre-term birth affects the risk of developing immune-mediated diseases. Front. Immunol..

[21] Pagano F (2021). Atopic manifestations in children born preterm: A long-term observational study. Children (Basel).

[22] Matías V (2012). Host and environmental factors influencing respiratory secretion of pro-wheezing biomarkers in pre-term children. Pediatr. Allergy Immunol..

[23] Kotecha S, Clemm H, Halvorsen T, Kotecha SJ (2018). Bronchial hyper-responsiveness in preterm-born subjects: A systematic review and meta-analysis. Pediatr. Allergy Immunol..

[24] Morata-Alba J, Romero-Rubio MT, Castillo-Corullón S, Escribano-Montaner A (2019). Respiratory morbidity, atopy and asthma at school age in pre-term infants aged 32–35 weeks. Eur. J. Pediatr..

[25] Hadchouel A (2018). Association between asthma and lung function in adolescents born very pre-term: Results of the EPIPAGE cohort study. Thorax.

[26] Grischkan J (2004). Variation in childhood asthma among former pre-term infants. J. Pediatr..

[27] Liu X (2014). Birth weight, gestational age, fetal growth and childhood asthma hospitalization. Allergy Asthma Clin. Immunol..

[28] Kalotas JO, Wang CJ, Noble PB, Wang KCW (2021). Intrauterine growth restriction promotes postnatal airway hyperresponsiveness independent of allergic disease. Front. Med..

[29] Kim HE, Song IG, Chung SH, Choi YS, Bae CW (2019). Trends in birth weight and the incidence of low birth weight and advanced maternal age in Korea between 1993 and 2016. J. Korean Med. Sci..

[30] Kim HS, Lee S, Kim JH (2018). Real-world evidence versus randomized controlled trial: Clinical research based on electronic medical records. J. Korean Med. Sci..

[31] Kim JA, Yoon S, Kim LY, Kim DS (2017). Towards actualizing the value potential of Korea health insurance review and assessment (HIRA) data as a resource for health research: Strengths, limitations, applications, and strategies for optimal use of HIRA data. J. Korean Med. Sci..

[32] Kim HS, Kim JH (2019). Proceed with caution when using real world data and real world evidence. J. Korean Med. Sci..

[33] National Health Insurance Sharing Service. Wonju-si: National Health Insurance Service. NHISS website https://nhiss.nhis.or.kr/bd/ay/bdaya001iv.do (2022).

[34] Korea Statistical Information Service. Daejeon: Statistics Korea. KOSIS website http://kosis.kr/statisticsList/statisticsListIndex.do?menuId=M_01_01&vwcd=MT_ZTITLE&parmTabId=M_01_01 (2022).

