# Supplementary figures and images for: Prevalence of asthma in preterm and associated risk factors based on prescription data from the Korean National Health Insurance database
Source: Sci Rep. 2023 Mar 18;13:4484. doi: 10.1038/s41598-023-31558-z (PMC10024678; doi:10.1038/s41598-023-31558-z)

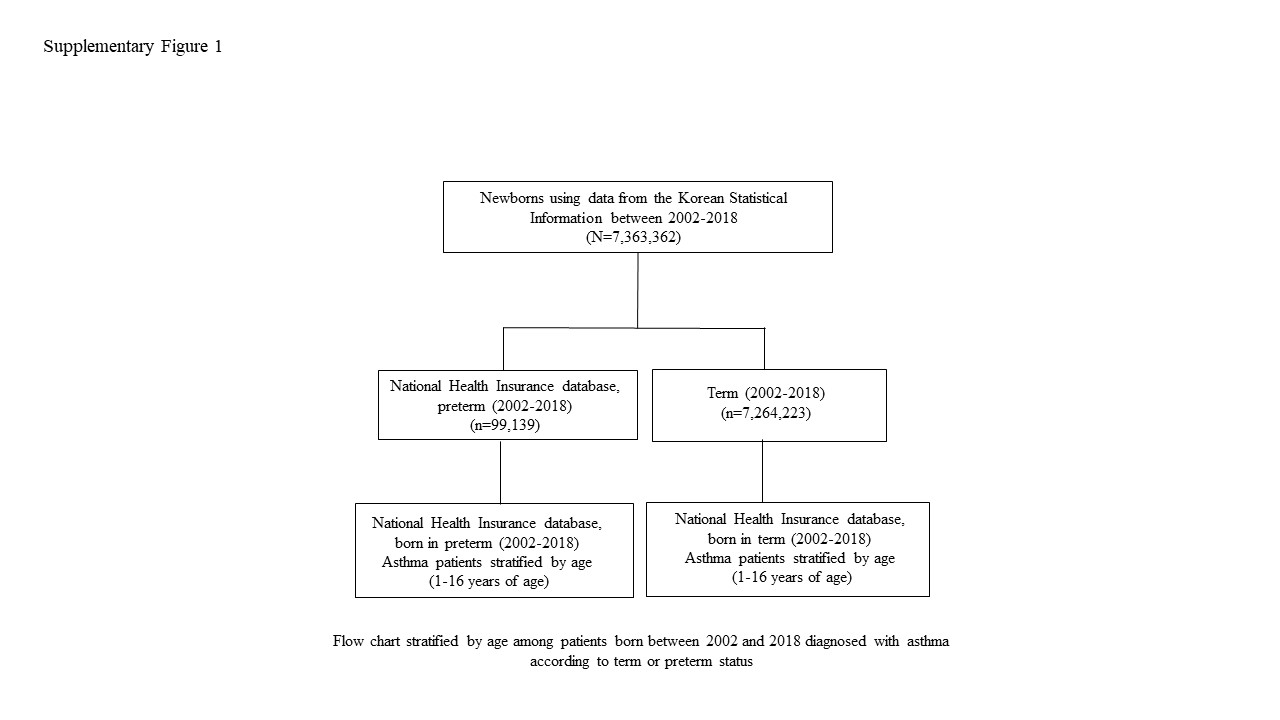

Supplement: Supplementary file 1 — Supplementary Information 1. [file 41598_2023_31558_MOESM1_ESM.jpg]
